# The role of NLRP3 inflammasome in digestive system malignancy

**DOI:** 10.3389/fcell.2022.1051612

**Published:** 2022-12-23

**Authors:** Cen-Cen Sun, Li Li, Hou-Quan Tao, Zhi-Chen Jiang, Liang Wang, Hui-Ju Wang

**Affiliations:** ^1^ Basic Medical Experimental Teaching Center, Zhejiang University, Hangzhou, China; ^2^ Key Laboratory of Gastroenterology of Zhejiang Province, Zhejiang Provincial People’s Hospital, Affiliated People’s Hospital, Hangzhou Medical College, Hangzhou, China; ^3^ Cancer Center, General Surgery, Department of Gastrointestinal and Pancreatic Surgery, Zhejiang Provincial People’s Hospital, Affiliated People’s Hospital, Hangzhou Medical College, Hangzhou, China; ^4^ Center for Plastic and Reconstructive Surgery, Department of Hand and Reconstruction Surgery, Zhejiang Provincial People’s Hospital, Affiliated People’s Hospital, Hangzhou Medical College, Hangzhou, China

**Keywords:** digestive system malignancy, pro-tumorigenic, anti-tumorigenic, NLRP3, inflammasome

## Abstract

Digestive system malignancies, the most common types of cancer and a major cause of death in the worldwide, are generally characterized by high morbidity, insidious symptoms and poor prognosis. NLRP3 inflammasome, the most studied inflammasome member, is considered to be crucial in tumorigenesis. In this paper, we reviewed its pro-tumorigenic and anti-tumorigenic properties in different types of digestive system malignancy depending on the types of cells, tissues and organs involved, which would provide promising avenue for exploring new anti-cancer therapies.

## Introduction

Digestive system malignancies, including gastric cancer (GC), hepatocellular carcinoma (HCC), colorectal cancer (CRC), pancreatic cancer (PC), *etc.*, are one of the main factors that endanger human health. According to statistics from the American Cancer Society, in 2022, the number of new cases of digestive system malignancies in the United States is expected to be 343,040 accounting for 18% of all incident cases, and the number of deaths will be 609,360 accounting for over one-half (28%) (Siegel et al., 2022). It is generally characterized by high morbidity, insidious symptoms and poor prognosis (Koc et al., 2020; Nagtegaal et al., 2020; Assarzadegan and Montgomery, 2021; Washington et al., 2021; Kushima, 2022). Therefore, early diagnosis, effective treatment and prognosis are the current focus of clinical prevention and treatment of digestive system tumors, which all need in-depth mechanism research as theoretical support.

Inflammasomes are a complex group of multiprotein complexes in the cytoplasm which could activate inflammation-associated caspases and induce the processing, maturation and secretion of the key pro-inflammatory cytokines, interleukin-1β (IL-1β) and IL-18, which thereby would initiate inflammatory responses, promote innate immune responses, and regulate acquired immunity (Watanabe et al., 2008; Schroder, 2010; Lamkanfi and Dixit, 2014; Guo et al., 2015; Broz and Dixit, 2016; Deets and Vance, 2021). Therefore, the inflammasome is also an important link and bridge between innate immunity and acquired immunity, and is considered to be the signal transduction center of the immune system.

**FIGURE 1:**
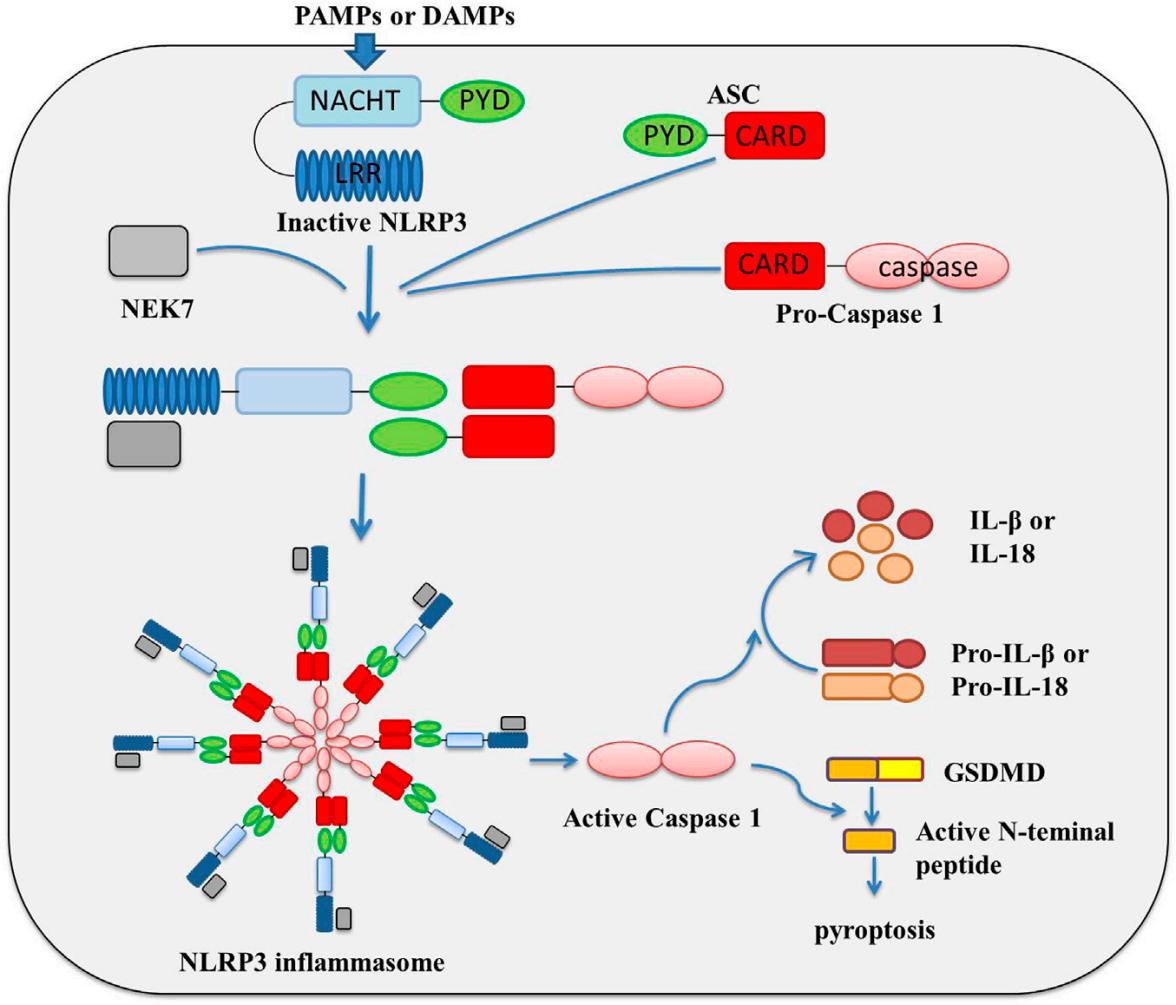
Activation and assembly Mechanism of NLRP3 inflammasome.

The inflammasome is composed of three key components: platform proteins, adaptor proteins and effector proteins. The classification of inflammasomes basically depends on the difference in platform proteins. At present, a variety of inflammatory complexes have been isolated and identified, including the Nod-like receptor (NLR) family pyrin domain containing protein 1 (NLRP1), NLRP3, NLR family CARD domain containing 4 (NLRC4), absent in melanoma 2 (AIM2) and RNA sensor RIG-I, *etc.*, among which, NLRP3 is the most detailed inflammasome studied at present (Schroder, 2010; Lamkanfi and Dixit, 2014; Guo et al., 2015; Broz and Dixit, 2016; Deets and Vance, 2021). NLRP3 inflammasome is composed of NLRP3 protein, adaptor apoptosis-associated speck-like protein (ASC) and the protein kinase NIMA related kinase 7 (NEK7), and effector pro-caspase-1 (Bae and Park, 2011; He et al., 2016). NLRP3 protein contains an N-terminal pyrin domain (PYD), a central NAIP, CIITA, HET-E, and TP1 (NACHT) domain, and a C-terminal leucine-rich repeats (LRRs) (Proell et al., 2008; Martinon et al., 2009). The activation and assembly of NLRP3 and other inflammasome are largely similar. Under normal condition, NLRP3 protein is thought to be auto-repressed by interaction of NACHT domain with LRRs; once it is activated upon pathogen-associated molecular patterns (PAMPs) or danger-associated molecular patterns (DAMPs), NLRP3 oligomerizes and triggers inflammasome assembly by recruiting ASC, pro-caspase-1 and NEK7 *via* homotypic domain interactions, involving NACHT, PYD, and CARD domain (Lamkanfi and Dixit, 2014; Martinon et al., 2009; Latz et al., 2013; Rahman et al., 2020) the assembly of the inflammatory complex realizes the aggregation of caspase 1, which greatly shortens the molecular distance, promotes the intermolecular self-cleavage of caspase 1, and then completes the activation of caspase 1 (Bae and Park, 2011; Shao et al., 2015; Swanson et al., 2019; Zhen and Zhang, 2019); activated caspase-1 causes the activation of inflammation-related transcription factors, and activation and releasing of inflammatory factors IL-1β and IL-18; in the meanwhile, by interaction with caspase-1, gasdermin-D (GSDMD) was cleaved and multimerized to form a peptide segment containing GSDMD, which could punch holes in the cell membrane, cause cell perforation, and induce pyroptosis (Figure 1) (Shi et al., 2015; Liu et al., 2017; Schneider et al., 2017; Wang et al., 2019; Chauhan et al., 2020; Downs et al., 2020; Karmakar et al., 2020; Shi et al., 2021a; Ding et al., 2021; Gao et al., 2021).

**FIGURE 2:**
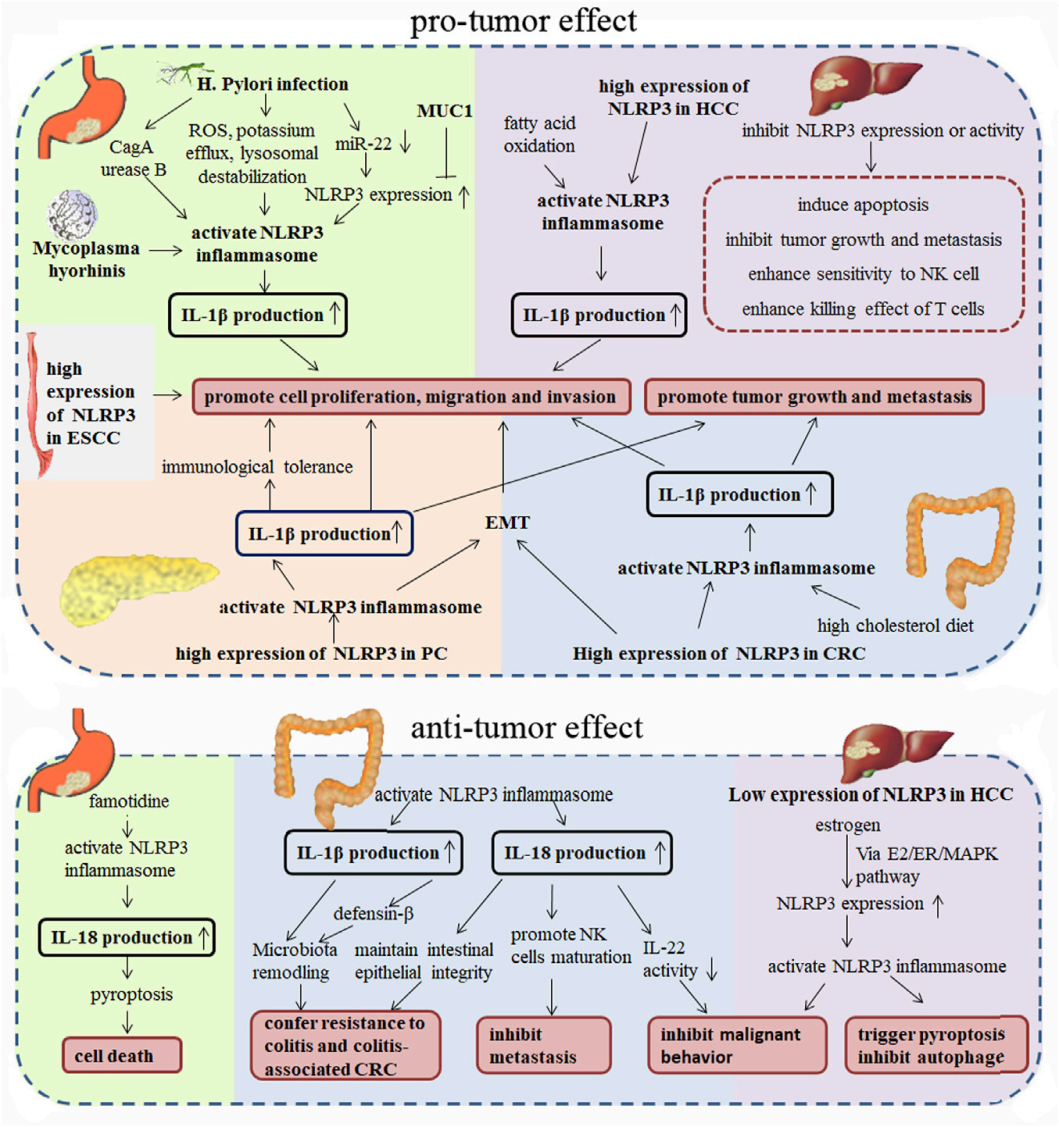
Pro-tumor and anti-tumor effect of NLRP3 in digestive system malignancies.

Many studies have shown that chronic inflammatory response was associated with a variety of tumors, and inflammation and persistent infection play important roles in different stages of tumor occurrence, development, malignant transformation, invasion, and metastasis (Oguma et al., 2010; Salem et al., 2018). The NLRP3 inflammasome, as an important intrinsic component of the human immune system, has attracted increasing attention in its functions in tumors. More and more studies have shown that NLRP3 inflammasome was closely associated with the progression of various tumors (such as gastric cancer (Castano-Rodriguez et al., 2014a; Castano-Rodriguez et al., 2014b; Zhang et al., 2022a), colorectal cancer (Dupaul-Chicoine et al., 2015; Perera et al., 2018; Shi et al., 2021b; Li et al., 2021; Marandi et al., 2021), liver cancer (Wei et al., 2014; Chen et al., 2020; Lee et al., 2021), *etc.*). NLRP3 is not only involved in the regulation of tumor itself, it also participates in the composition of the tumor microenvironment, and has dual effects of promoting and inhibiting tumorigenesis in different tissues or cell types. In this article, we review the research status of NLRP3 in digestive system malignancies (Figure 2), which might provide theoretical basis for the potential future clinical applications.

### The research of NLRP3 in gastric cancer

Gastric cancer is one of the most common malignant tumors, and chronic infection with H. pylori is believed to be the most important risk factor for the development of gastric cancer (about 75% of gastric cancers were associated with it in previous research) (Correa, 2013; Wang et al., 2014; Plummer et al., 2016; Van Cutsem et al., 2016; Molina-Castro et al., 2017; Engstrand and Graham, 2020; Smyth et al., 2020; Thrift and El-Serag, 2020). Several researches have demonstrated that H. pylori infection could induce ROS production, potassium efflux and lysosomal destabilization, which would finally lead to activation of NLRP3 inflammasome (Kim et al., 2013; Semper et al., 2014; Koch et al., 2015; Li et al., 2015; Perez-Figueroa et al., 2016; Man, 2018). Further studies have shown that the cag pathogenicity island (cagPAI) and urease B subunit (the H. pylori virulence factors) could promote the IL-1β secretion by potentiating activation of the NLRP3 inflammasome in immune cells, and excessive production of IL-1β were considered to be extensively linked to gastric carcinogenesis (Kim et al., 2013; Semper et al., 2014; Koch et al., 2015). In recent research, Zhang et al. found that cytotoxin-associated gene A (CagA) encoded on the cagPAI of H. pylori could activate the NLRP3 inflammasome, promote the secretion of IL-1β and IL-18 secretion, and finally promote cell proliferation, migration and invasion in gastric cancer (Zhang et al., 2022a). Li et al. found that H. pylori infection could inhibit the expression of miR-22 in gastric epithelial cells, which would upregulated the expression of NLRP3 and enhance H. pylori-induced gastric carcinogenesis (Li et al., 2018). And Ng et al. found that MUC1 is an important negative regulator of NLRP3 inflammasome which could protect epithelial barrier from H. pylori infection by negative regulating NLRP3 expression *via* a nuclear factor (NF)-κB-dependent pathway (Ng et al., 2016). Moreover, Mycoplasma hyorhinis, which was detected in 56% of gastric cancer, was also found to be able to active NLRP3 inflammasome and induce IL-1β secretion, thus promote gastric cancer cell migration and invasion (Xu et al., 2013). Besides, Huang et al. reported that in gastric cancer cells, famotidine, a gastric antisecretory drug, could promote the activation of NLRP3 inflammasomes by upregulating expression of NLRP3, ASC, and Caspase-1, then enhance IL-18, not IL-1β, mature and secretion, thus trigger cell pyroptosis and aggravate inflammation, which was considered to be critical in development of gastric cancer (Huang et al., 2021). However, according to the study of West et al., in gp130 ^F/F^ mouse model, NLRP3 expression levels did not affect the development of gastric tumors, and the cellular processes associated with tumorigenesis in the gastric mucosa, such as proliferation, apoptosis, and inflammation did not changed in the NLRP3 knockout gp130 mouse, which suggested that NLRP3 might not play a major role in promoting inflammasome-driven gastric tumorigenesis (West et al., 2022).

### The research of NLRP3 in hepatocellular carcinoma

NLRP3 is one of the most widely studied NLR family members in liver disease, which was widely distributed in parenchymal and non-parenchymal hepatocytes. NLRP3 was reported to play essential roles in the pathogenesis of alcohol-associated liver disease, non-alcoholic fatty liver disease/non-alcoholic steatohepatitis, cirrhosis and fibrosis, which ultimately leads to hepatocellular carcinoma (HCC) (Wei et al., 2014; Wree et al., 2014; Mridha et al., 2017; Luan et al., 2018; Pourcet et al., 2018; Chen et al., 2020; Tao et al., 2020; Xie et al., 2020; Surlin et al., 2021). Many studies have shown that the NLRP3 inflammasome has a promoting effect on HCC. NLRP3 has been found highly expressed in HCC tissues, and inhibition of NLRP3 inflammasome activation could induce apoptosis and regulate the levels of inflammatory factors, which would suppress the growth of HCC cells (Chen et al., 2022) (Li et al., 2020a). Knockout of NLRP3 in HCC cells also inhibited tumor growth and metastasis *in vivo*, as well as increased the sensitivity to NK cell cytotoxicity (Lee et al., 2021). While enhanced NLRP3 inflammasome activation and IL-1β secretion by fatty acid oxidation in M2 macrophages could enhance the proliferation, migration, and invasion of HCC cells (Zhang et al., 2018). Besides, Ding et al. found that inhibition of NLRP3 in Hep3B cells promoted the killing effect of T cells to cancer cells by repressing the expression of immune checkpoints (Ding et al., 2022). And in Sonohara’s research, the adjacent tissue of HCC was reported to be in a hyper-inflammatory state due to the overexpression of NLRP3, NLRC4, and AIM2 genes, which was showed to be closely related to poor overall survival, suggesting that the high expression of NLRP3 may become an independent prognostic factor for overall survival after surgery in HCC (Sonohara et al., 2017). However, there are a few studies indicated the protective role of NLRP3 inflammasome in HCC progression. Wei et al. (2014) found that the expression of NLRP3 inflammasome components was down-regulated in HCC tissues and inversely correlated with pathological grades and clinical stages; the up-regulation of NLRP3 inflammasome expression mediated by estrogen through the E2/ER/MAPK pathway, could significantly inhibit the malignant behavior of HCC cells (Wei et al., 2015; Wei et al., 2019). Therefore, the controversial role of NLRP3 in HCC needs more research and data to explore and clarify.

### The research of NLRP3 in colorectal cancer

Colorectal cancer (CRC) is the most common malignant tumor of digestive system, and its mortality rate ranks second in the world (Siegel et al., 2022). Meanwhile, inflammatory bowel disease (IBD), including ulcerative colitis and Crohn’s disease, has been identified as a high-risk factor for CRC (Dekker et al., 2019). Therefore, inflammasome, as an important link of inflammatory response, plays an important role in the occurrence and development of CRC (Dupaul-Chicoine et al., 2015; Deng et al., 2019; Shao et al., 2020; Shi et al., 2021b; Li et al., 2021; Marandi et al., 2021).

Research on the NLRP3 inflammasome in colorectal tumors is mainly focused on its function in colitis-associated CRC, and it is controversial. It was reported that NLRP3 may confer protection against colitis and colitis-associated tumorigenesis, by mediating the secretion of IL-18, which could promote intestinal epithelial cell differentiation, maintain intestinal epithelial integrity, and reduce intestinal epithelial cell proliferation during colitis remission; NLRP3 deficient mice were found to be more susceptible to dextran sodium sulfate (DSS) and 2,4,6-trinitrobenzenesulfonic acid, and showed more sever colonic inflammation, and higher carcinogenesis (Allen et al., 2010; Zaki et al., 2010; Zaki et al., 2011). Hirota et al. also found that knockout of NLRP3 resulted in altered expression of colonic defensin-β, decreased antimicrobial secretions, and unique gut microbiota types change, which finally reduced intestinal resistance to enteric pathogens (Hirota et al., 2011). And Yao’s research indicated that hyperactive NLRP3 enhances IL-1β but not IL-18 secretion, leading to gut microbiota remodeling and regulatory T cells (Tregs) induction, thus confers disease resistance (Yao et al., 2017). Recently, Dupaul-Chicoine et al. reported that the NLRP3 inflammasome could reduce the occurrence of enteritis-related CRC and inhibit metastatic growth of CRC in liver (Dupaul-Chicoine et al., 2015). This study also found that the activation of IL-18 mediated by NLRP3 could enhance tumoricidal activity of NK cells, which directed to the metastasized colonic tumor cells in the mouse liver (Dupaul-Chicoine et al., 2015). Moreover, IL-18 also could fine-tune the biological activity of IL-22 *via* inhibit the production of the IL-22 binding protein, And IL-22 was reported to promote tumor development at later stages (Huber et al., 2012).

However, in contrast to the aforementioned studies, NLRP3 inflammasome also showed the potential to promote carcinogenesis and cancer progression in CRC. Bauer et al. showed that NLRP3 gene knockout would alleviate DSS-induced colitis (Bauer et al., 2010). Besides, high cholesterol diet induced colon carcinogenesis was mediated by NLRP3 inflammasome activation (Du et al., 2016a). Moreover, NLRP3 was found highly expressed in CRC tissues and mesenchymal-like CRC cells, and promoted epithelial-mesenchymal transition (EMT) in an inflammasome-independent way; knockdown of NLRP3 in CRC cells decreased cell proliferation, migration and invasion ability (Wang et al., 2016; Shao et al., 2020; Shi et al., 2021b; Marandi et al., 2021). Furthermore, macrophages-CRC cell crosstalk activated NLRP3 inflammasomes and induced IL-1β secretion in macrophages, thus promoted the invasion and migration of CRC cell, and drive metastasis to the liver (Deng et al., 2019; Zhang et al., 2022b). The controversy maybe due to NLRP3’s broad activity in intrinsic properties and microenvironments of a tumor cell, and further studies are needed to explain the diversity of its functions.

### The research of NLRP3 in pancreatic cancer

In the pancreas, NLRP3 activation was found to be necessary for the development of experimental acute pancreatitis, pancreatic fibrosis, obesity-induced insulin resistance, and to be a critical inflammatory mechanism in pancreatic cancer (Vandanmagsar et al., 2011; Wan et al., 2016; Boone et al., 2019; Li et al., 2020b; Das et al., 2020; Sendler et al., 2020; Tang et al., 2020; Gao et al., 2021). In Liu’s study, NLRP3 was reported to be upregulated in pancreatic ductal adenocarcinoma (PDA), as shown in both infiltrating immune cells and tumor cells; and the activation of NLRP3 inflammasome in macrophages would induce immunological tolerance and promote the tumor growth (Liu et al., 2020). Boone et al. found that the platelet NLRP3 inflammasome is upregulated in a murine model of pancreatic cancer which would promote platelet aggregation and tumor growth (Boone et al., 2019). Daley et al. (2017) reported that the activation of NLRP3 inflammasome in macrophages directs tolerogenic T cell differentiation, which would induce immunological tolerance and promote pancreatic tumor growth. Hu et al. (2018) showed that down-regulated NLRP3 signaling in pancreatic cancer cells would suppress progression of pancreatic cancer and decrease epithelial-mesenchymal transition-induced cell invasion. In short, NLRP3 could promote the tumor growth, progression and cell invasion in pancreatic cancer.

### The research of NLRP3 in other cancers

The roles of NLRP3 in other digestive system malignancy were constantly being discovered. Yu et al. (2022) found that the level of NLRP3 inflammasome was significantly up-regulated in human esophageal squamous cell carcinoma (ESCC) tissues, which was positively correlated with tumor malignancy. And the knockdown or overexpression of NLRP3 could markedly abolish or promote cell migration and invasion and decrease or increase cell mobility, respectively, which suggested that NLRP3 inflammasome-mediated inflammation could contribute to the development and progression of ESCC (Yu et al., 2020). Matsushita et al. showed that lower expressions of NLRP3 in primary sclerosing cholangitis (PSC) patients were associated with the development of cholangiocarcinoma (Matsushita et al., 2015). Overall, the diverse roles of NLRP3 inflammasome in different digestive system tumors still need to be confirmed and explored, and its related mechanism needs to be further studied.

### The NLRP3 inflammasome as a promising target in cancer therapy

The involvement of NLRP3 inflammasome in inflammation-related diseases and cancers makes it an attractive therapeutic target. Many efforts have been made to explore effective compounds that can inhibit the NLRP3 inflammasome components, and various agents have been developed, including MCC950, CY-09, Fluoxetine, Erianin, INF39, Parthenolide and BAY 11-7082, Oridonin, RRx-001, Manoalide, Pristimerin, Piperlongumine, OLT1177, Celastrol, Tranilast, and so on (Table 1).

**TABLE 1:** inhibitors of NLRP3 inflammasome.

Drugs	Mechanism	Refernce
MCC950	binds to Walker B and partially Walker A motif within the NLRP3 NACHT domain to block ATP hydrolysis	Coll et al. (2015); Jiang et al. (2017); Coll et al. (2019); Mekni et al. (2019); Tapia-Abellan et al. (2019)
CY-09	binds to Walker A motif within NLRP3 NACHT domain, thus inhibits NLRP3 ATPase activity	Jiang et al. (2017)
Fluoxetin	binds to the NLRP3 NACHT Domain, thus prevents NLRP3-ASC inflammasome assembly	Ambati et al. (2021), (Du et al., 2016b)
Erianin	associates with the Walker A motif in the NACHT domain and suppresses NLRP3 ATPase activity	Zhang et al. (2021a)
INF39	inhibits the ATPase activity of NLRP3	Shi et al. (2021c)
Parthenolide and BAY 11-7082	inhibits the ATPase activity of NLRP3	Juliana et al. (2010)
Oridonin	binds to cysteine 279 of NLRP3 NACHT domain to block NLRP3–NEK7 interaction	He et al. (2018)
RRx-001	binds to cysteine 409 of NLRP3 NACHT domain to block the NLRP3-NEK7 interaction	Chen et al. (2021a)
manoalide	binds to Lys 377 of the NLRP3 NACHT domain to block the NEK7 and NLRP3 interaction	Li et al. (2022a)
Pristimerin	binds to NLRP3 NACHT domain to block the NEK7 and NLRP3 interaction	Zhao et al. (2021)
Licochalcone B	binds to NEK7 to block the NEK7 and NLRP3 interaction	Li et al. (2022b)
Piperlongumine	block the interaction between NEK7 and NLRP3, and suppress the assembly of the NLRP3 inflammasome	Shi et al. (2021d)
Dapansutrile (OLT1177)	prevents NLRP3-ASC and NLRP3-caspase-1 interaction, thus inhibiting NLRP3 inflammasome oligomerization	Marchetti et al. (2018)
Celastrol	interrupts ASC oligomerization and NLRP3 complex formation	Sang et al. (2018), Yu et al. (2017)
Tranilast	binds directly to the NACHT domain of NLRP3, and inhibit NLRP3- NLRP3 interaction	Huang et al. (2018)
Tubocapsan olide A	bound to the cysteine 514 residue (Cys514) of NLRP3, inhibited the activation of NLRP3 inflammasome	Chen et al. (2021b)

MCC950/CRID3/CP-456773 is the most studied selective inhibitor of NLRP3 inflammasome. MCC950 directly binds to a region crossing the Walker B and partially Walker A motifs within the NACHT domain of NLRP3, thereby blocks ATP hydrolysis and prevents NLRP3 oligomerization and activation (Coll et al., 2015; Coll et al., 2019; Mekni et al., 2019; Tapia-Abellan et al., 2019). CY-09, fluoxetine and Erianin all show the ability to directly bind to the ATP-binding motif of NLRP3 NACHT domain to inhibits NLRP3 ATPase activity and suppresses NLRP3 assembly (Du et al., 2016b; Jiang et al., 2017; Ambati et al., 2021; Zhang et al., 2021a). Besides, INF39, Parthenolide and BAY 11-7082 are also able to inhibit the ATPase activity of NLRP3, however, the detailed molecular mechanism is not yet fully understood (Juliana et al., 2010; Shi et al., 2021c).

Oridonin, RRx-001, manoalide, and pristimerin, are showed to supress the assembly and the activation of the NLRP3 inflammasome by covalently binding to the NACHT domain to block the NLRP-NEK7 interaction (He et al., 2018; Chen et al., 2021a; Zhao et al., 2021; Li et al., 2022a). While Licochalcone B is found to directly bind to NEK7 and inhibit the interaction between NLRP3 and NEK7, thus inhibit the activation of NLRP3 inflammasome (Li et al., 2022b). Piperlongumine also can suppress assembly of the NLRP3 inflammasome by interrupting NLRP3 and NEK7 interaction (Shi et al., 2021d).

OLT1177/Dapansutrile is able to prevent NLRP3-ASC and NLRP3-caspase-1 interaction, thus inhibited NLRP3 inflammasome oligomerization (Marchetti et al., 2018). Celastrol may inhibit NLRP3 inflammasome activation *via* interrupting of ASC oligomerization and subsequently NLRP3 inflammasome assembly (Yu et al., 2017; Sang et al., 2018). Tranilast, an old anti-allergic clinical drug, can directly bind to the NACHT domain of NLRP3 to block NLRP3-NLRP3 interaction and the subsequent ASC oligomerization (Huang et al., 2018). Tubocapsan olide A can bind to the Cys51) of NLRP3 to inhibit the activation of NLRP3 inflammasome (Chen et al., 2021b).

These NLRP3 inflammasome inhibitors exhibit efficient anti-inflammatory effect in inflammation-related diseases. Moreover, some studies also have shown that some of them even possess potent anti-tumor effect, such as MCC950, Erianin, Oridonin, RRx-001, Pristimerin, which were found able to suppress cell proliferation and migration, induce apoptosis, and delay tumor growth (Scicinski et al., 2015; Huang et al., 2017; Li et al., 2020c; Yaw et al., 2020; Shi et al., 2021b; Zhang et al., 2021b; Li et al., 2022a; Ding et al., 2022; Hong et al., 2022). MCC950, MCC950, Erianin, Oridonin and RRx-001 also showed ability to reshape the immune microenvironment by increasing the number of effector T cells and decreasing immunosuppressive cell accumulation (Brzezniak et al., 2016; Huang et al., 2017; Guo et al., 2020; Yang et al., 2021). These results indicate that NLRP3 inflammasome inhibitors have great promise as a novel cancer therapeutic agent.

## Conclusion and perspective

In this review, we provided a brief overview of the biological importance of NLRP3 inflammasome in different forms of digestive system malignancy. In gastric, pancreas and esophageal cancer, NLRP3 inflammasome mainly act as a tumor promoter to regulate the tumor growth, invasion and metastasis, and so on. In cholangiocarcinoma and liver cancer, NLRP3 inflammasome could inhibit the tumor progression, and in colorectal tumor, it might have the dual effect of promoting and inhibiting. It was believed that the pro-tumorigenic and anti-tumorigenic properties of NLRP3 inflammasome are largely determined on effector cytokines (IL-1β and IL-18) and the types of target cells, tissues and organs involved. Although IL-1β and IL-18 belong to IL-1 family, they paly different roles in tumor biology and tumor immunity. Many studies have demonstrated that IL-1β can enhance tumor progression and resistance to cancer therapy; while IL-18 exerts an anti-tumor activity, as it can stimulate anti-tumor immunity by activating NK, Th1, CD4^+^ T cells, and CD8^+^ T cells (Sharma and Kanneganti, 2021; Sun et al., 2022). Besides, we also summarized the recent advances in specific inhibitors of NLRP3 inflammasome and their potential in cancer therapy.

In conclusion, NLRP3 inflammasome played a significant part in occurrence and development of various digestive system tumors, which requires more in-depth and comprehensive research in the future. And the biological relationship between NLRP3 inflammasomes and cancer would might open up new directions for anti-cancer therapies.
